# Becoming partners, retaining autonomy: ethical considerations on the development of precision medicine

**DOI:** 10.1186/s12910-016-0149-6

**Published:** 2016-11-04

**Authors:** Alessandro Blasimme, Effy Vayena

**Affiliations:** 1 Health Ethics and Policy Lab, University of Zurich, 84 Hirshengraben, 8001 Zurich, Switzerland; 2 U1027 Inserm – Université Paul Sabatier, Toulouse, France

**Keywords:** Precision medicine, Participation, Ethics, Autonomy, Personalized medicine

## Abstract

Precision medicine promises to develop diagnoses and treatments that take individual variability into account. According to most specialists, turning this promise into reality will require adapting the established framework of clinical research ethics, and paying more attention to participants’ attitudes towards sharing genotypic, phenotypic, lifestyle data and health records, and ultimately to their desire to be engaged as active partners in medical research.

Notions such as participation, engagement and partnership have been introduced in bioethics debates concerning genetics and large-scale biobanking to broaden the focus of discussion beyond individual choice and individuals’ moral interests. The uptake of those concepts in precision medicine is to be welcomed. However, as data and medical information from research participants in precision medicine cohorts will be collected on an individual basis, translating a participatory approach in this emerging area may prove cumbersome. Therefore, drawing on Joseph Raz’s perfectionism, we propose a principle of *respect for autonomous agents* that, we reckon, can address many of the concerns driving recent scholarship on partnership and public participation, while avoiding some of the limitations these concept have in the context of precision medicine. Our approach offers a normative clarification to how *becoming* partners in precision is compatible with retaining autonomy.

Realigning the value of autonomy with ideals of direct engagement, we show, can provide adequate normative orientation to precision medicine; it can do justice to the idea of moral pluralism by stressing the value of moral self-determination: and, finally, it can reconcile the notion of autonomy with other more communitarian values such as participation and solidarity.

## Background

Personalized medicine refers to a family of approaches in biomedical research and clinical practice aimed at “steering the patient to the right drug at the right dose at the right time” [1]. This idea has always enjoyed some recognition in modern medicine. However, in the last couple of decades, it almost reached the status of a distinctive way of thinking about health, disease and therapy. Since the early days of pharmacogenetics, and up until the announcement of the Precision Medicine Initiative (PMI) in January 2015, this emerging paradigm has been promising nothing less than a revolution in medicine. However, precision medicine does not only promise to change our understanding of disease and the way health care is delivered to patients. It also contains an element of novelty with respect to how research participants and patients involved in its clinical development should be considered, what they should be entitled to, and how they should be treated as they get associated with the enterprise of precision medicine. In particular, the emergence of precision medicine is accompanied by a demand for a participatory understanding of the role of patients and research participants as “active partners in clinical research” [2].

Interestingly, this language is not entirely new. In the early Fifties, at the Clinical Center of the National Institutes of Health’s, intramural research had to comply with a set of ethical principles (remarkably advanced for that period) that defined research participants as partners in research [3]. Thirty years later, in a 1984 paper, Daniel Callahan recognized that the raise of bioethics had “brought patients into a full *partnership* with physicians in their medical care” ([4], added emphasis).^1^

Despite such appearances, however, the notion of partnership has not enjoyed much consideration in mainstream bioethics until relatively recent times. In truth, this notion is often presented as a way to cope with the shortcomings stemming from “the paramount position of the individual in ethics” [5]. Based on this intuition, many in bioethics started to emphasize the importance of a broader set of normative concerns having to do with considering research subjects as partners who should be given ways to participate in the governance of research.

Similar appeals to partnership, participation and engagement^2^ characterize the normative framing of precision medicine by the leadership of the Precision Medicine Initiative.

Launched in January 2015 by President Barack Obama, the Precision Medicine Initiative revolves around the creation of a large national cohort (run by the National Institutes of Health) of at least one million individuals who will contribute extensive amounts of medical, genetic, genomic, behavioral, phenotypic and biomarkers data, as well as lifestyle and other personal information. Research participants will continue to contribute data over the years, thus enabling researchers to look for medically relevant correlations based on an ever-growing longitudinal cohort. Thanks to rapid progress in data science, machine learning and big data analytics, the precision medicine cohort is expected to usher in a new era of biomedical research – thus leading to new “prevention and treatment strategies that take individual variability into account” [2]. Precision medicine is premised on the idea that the “patient is an enormous repository of information that needs to be harvested as a partnership not only in clinical care but in discovery [as a way to] define wellness and its progression to disease, rather than traditional medicine that defines disease and its progression to death” [6]. If the promise of precision medicine will materialize, there will be considerable improvements in understanding disease risk, individual predispositions, as well as the contribution of the environment and lifestyle to disease onset and to the way individuals respond to therapy. This is expected to result in a considerably increased capacity to tailor treatment and prevention strategies to the needs and characteristics of individuals – thus improving treatment outcomes and making health care systems more efficient and more sustainable.^3^

Interestingly, for the establishment of the precision medicine cohort, the PMI explicitly embraces ideals of participation and partnership, as it sets out to capitalize on “Americans’ growing desire to be active partners in modern science” [2].

## Discussion

### The normative construction of research partners

In research ethics, concepts such as partnership and participation designate a procedural ideal of direct engagement of research subjects in the organization and implementation of studies in which they are enrolled.

Interestingly, the ethical values that underpin this ideal are often constructed to supplement the centrality of individual moral interests that, since the late Seventies, has been characteristic of clinical research ethics and medical ethics.

Such novel normative orientation gradually emerged in the Nineties, in concomitance with the growth of large-scale research biorepositories (commonly known as biobanks). More recently, technical progress in genome sequencing gave further impetus to both research and large-scale biobanking initiatives – such as the constitutions of national biological repositories [7]. This type of biological collection, many argued, pose novel ethical challenges. In particular, it has been argued that biobanking promoted a shift in focus from individuals to groups. What these repositories typically collect is biological material from genetically isolated populations, populations bearing medically favorable traits (like longevity or reduced incidence of a given disease), minorities, families in which a given disease shows up with increased frequency, groups of people affected by the same conditions or sharing a common genetic mutation, and patients affected by a rare or orphan disease. As a consequence, in that period, bioethical debates started to consider novel issues, such as the rights and interests of those who contribute biological material to research as members of a particular group.

For instance, when culturally sensible issues are at stake, collective forms of authorization (or group consent) can serve morally relevant interests that it would otherwise be hard to take into account [8]. Moved by similar concerns, some have also observed that “American bioethics has been dominated by the goal of individual control” (epitomized by the emphasis on individual consent), and that forms of deliberative engagement in the governance of large scale genomics research may broaden the scope of discussion in favorable ways [9].

This focus on collectivities is also visible in discussions about returning research benefits to the community that contributed tissue and information, and thus made research possible in the first place. This entails soliciting communities of research participants to express their views and expectations concerning a given study, taking historical and cultural issues in due consideration and meeting the local needs of research communities as a way to give recognition to their contribution [8]. Similar considerations are at play also in recent discussions concerning next generation sequencing research. Most experts agree that, in this domain, dedicated procedures should be in place to return incidental findings and research results to participants [9–14]. However, there is growing recognition of the possibility that return policies could be explored and negotiated in a collective fashion before sample, data and information are collected and analyzed [15]. Partnership, in this case, would grant a more active role to research subjects, patients and communities as to their capacity to have a say on a number of issues, ranging from protocol design to criteria for recruitment, from feedback of research findings to direct access to data and assessment of results.

For these strains of scholarship, the individual is no longer the only subject of moral rights – and thus the privileged object of moral consideration. Groups, communities and populations have cultural, identity-shaping interests and should also be afforded appropriate moral consideration. In this sense, valuing participation amounts at finding ways to give a voice to those groups by involving them in decisions concerning the collection, analysis and circulation of their samples and data. Following this trend, a whole constellation of values such as reciprocity, mutuality, solidarity, universality and citizenry has taken center stage, informing what has been described as a communitarian turn in bioethics [5, 16].

Finally, *transparency* and *accountability* are often considered as corollary values to direct engagement and are routinely appealed to as ways to create and maintain public trust in the scientific enterprise and its governance structures [17–26].

It should now be clear that participation and partnership refer to a procedural ideal of direct engagement in the activities that pertain health research and the development of novel health paradigms. Advocates of partnership view direct engagement as a way to overcome the alleged limitations of previously established ethical frameworks and procedures focused almost exclusively on respect for individual choice and autonomy in biomedical research and clinical practice.

### Partnership, participation and precision medicine

Ideals of partnership, participation and the other core values associated with them are routinely recalled in discussions about an increasingly active role of research participants in the development of personalized and precision medicine [1, 27–30].

This feature should be greeted as potentially enriching the scope of ethical discussion in this novel domain. However, from these appeals, it is not entirely clear how a national precision medicine cohort of one million individuals could integrate those ideals in a meaningful way. For one thing, research participants will join the cohort on a voluntary and, most probably, as individuals, that is, not as members of a particular group. Moreover, even if patient groups or any other types of communities decided to join the cohort, their communitarian interests would have to coexist with those of other (potentially many) groups and communities that may decide to do the same. In other words, the kind of communitarian bonds that a participatory ethos is supposed to capture and to promote are either yet to be formed or bound to compete for the opportunity to have a say regarding the governance of the cohort. This has so far surprisingly escaped scholarly attention.

Normative emphasis on public participation and partnership at this early stage needs to come to terms with the fact that a precision medicine cohort will initially be more of a collection of individual data than a truly collective entity. Given its large size, it will take time to assemble a cohort in the first place, and, as a consequence, the community bonds that tie research participants together will also only be forming gradually along the way. Moreover, according to many, recruitment will likely continue even after the threshold of one million participants is reached, thus making the communitarian contours of the cohort somewhat even more elusive to capture.

Obviously, the same holds for large-scale precision medicine cohorts that, in the near future, will be constituted outside of the United States, in all other countries that will endorse precision medicine as a promising domain of innovation.

It follows that, as such cohorts get assembled, public participation and collective engagement will not be as meaningful to precision medicine research subjects as they clearly are in the case of population genetics biobanks. Nevertheless, we think this should not entail a fallback towards an individualistic framing of the ethical stakes of precision medicine. As a precision medicine cohort takes shape, research participants can still be offered meaningful ways to fulfill the interests that participation and partnership are supposed to protect. In particular, perspective participants should be offered ways to *become members* of communities and groups that will form in the future, once the cohort acquires a more definite shape. In other words, there can be good ethical reasons to enable participants who enroll in precision medicine cohorts to *become partners* – even if we look at them mainly as autonomous individuals. To this aim, we propose a richer understanding of the idea of autonomy that might bear on the process of enrolment of research participants in precision medicine cohorts.

### Autonomy revisited

In moral and political philosophy, the value of personal autonomy expresses a concern for the idea “that people should make their own life” [31]. Such concerns focus on ideals of personal self-governance (or personal self-rule) and freedom from external control and undue interference, and points to the ethical worth of autonomous *agents*.

To begin with, we can spell out our version of the principle of respect for autonomy as *respect for autonomous agents* (or concern for *autonomous lives*), rather than respect for autonomous action or choice. Focusing on autonomous *choice* is at the core of informed consent intended as a form of autonomous authorization (to perform medical activities on one’s body in the context of clinical care or research). This understanding of autonomy has long been prominent in bioethics: it stresses the value of being adequately informed, the importance of the capacity to process this information, and the absence of coercion or manipulation.

Indeed, valuing individual choice is of the utmost importance in the context of clinical care and medical research. However this understanding of autonomy may prove too narrow. Following moral philosopher Joseph Raz, we maintain that “one is autonomous if one determines the course of one’s life by oneself” [31]. Obviously, this does not imply that one can gain full control of his or her life without any influence from other people. What this account of autonomy says is just that being coerced into one’s own decisions or acting while being the object of manipulation by others is not compatible with the ideal of an autonomous life. As a consequence, third parties have a prima facie duty not to unduly interfere with one’s efforts at shaping the course of one’s life from a moral point of view.

According to Raz, however, the absence of coercion and manipulation is a necessary but not sufficient condition for an autonomous life absent “an adequate range of options for [a person] to choose from” [31].^4^ This further assumption follows from considering autonomy as a guiding moral ideal for people’s life (as opposed to considering it as an attribute of specific actions). This has a consequence and a corollary. The consequence of thinking that autonomy entails a meaningful set of options is that it creates positive duties to actually provide those options. Obviously, this applies more directly to those individuals or institutions that are capable of influencing the availability of moral options at a societal level; but Raz does not exclude that each of us who is committed to the value of autonomy also carries this duty in one way or another. The corollary of that is moral pluralism. What this expression designates is “the view that there are various forms and styles of life which exemplify different virtues and which are incompatible” (in the sense that they cannot be pursued at the same time by the same person) [31]. According to Raz, autonomy “requires that many morally acceptable, though incompatible, forms of life be available to a person” [31], and therefore it entails moral pluralism.^5^

For the purposes of our argument, we focus in particular on the most characteristic theoretical component of a Razian understanding of autonomy, that is, the importance of a *meaningful range of worthwhile options.* 6 According to this criterion, having more options, per se, does not enhance one’s capacity to be autonomous. For example, having the option of doing something morally bad (like stealing a drug), or being able to choose between meaningless alternatives (e.g. choosing the color of the pills we are asked to take if we are enrolled in a clinical research study) cannot be said to enhance one’s autonomy.^7^ A meaningful range of worthwhile options in the context of health research has to do with morally valuable interests that one has as a research participant. Some of those interests are normally ensured at the moment of ethical assessment of a given research project by a research ethics committee (REC) or institutional review board (IRB). This is the case, for instance, of interests that pertain to physical integrity, like being exposed to the minimum possible risk of physical harm, or other valuable states, like avoiding both direct and indirect privacy violations. But the attainment of other valuable states of being cannot be delegated entirely to RECs or IRBs. Those states do not pertain to the sphere of protection – like those just mentioned – but to the sphere of freedom. While the former depend on the existence of independent oversight mechanisms, the latter depend instead on the availability of options to choose from. For instance, being given the option of participating in decisions about the governance of a research cohort, is typically assumed to be a valuable thing by scholars who support a participatory framing of research ethics. According to our account of autonomous agency, it is relevant to personal autonomy that one is offered the opportunity to be engaged directly in decisions of this kind. Direct engagement of this sort is valued by individuals as it affords the freedom to act in a certain way, namely as members of a self-governing community that tries to attain what is of common interest. Having the option of assuming this role is valuable for our Razian interpretation of autonomy.

### Becoming partners

We are sympathetic to the general idea that progress in precision medicine is predicated upon the recognition of research participants as active partners. This ethical stance is recurrently coupled with calls for supplementing the ethical framework developed in the aftermath of the Belmont Report and its emphasis on autonomous choice.

But the analytic point we would like to make here is that the idea of partnership – and the concerns it sets out to address – can find in our Razian understanding of autonomy a valid normative ally. As we said above, a major issue with the creation of large national precision medicine cohorts is that participants do not initially constitute a community of the kind that ideals of partnership and participation are supposed to serve. It follows that, if the kind of freedom those ideals promote is indeed ethically valuable, participants should be offered meaningful options to *become* members of such a community, and thus to have access to those freedoms. We reckon that valuing those freedoms entails making them accessible to choice – which falls within our Razian interpretation of autonomous agency.

But how does respect for autonomous agents play out in practice? What does this principle require? And who should be paying attention to the ethically relevant interests it promotes? We think the domain in which the principle matters the most is in the design of participatory governance structures for precision medicine cohorts.

More specifically, at the moment of enrollment, participants should be presented with a detailed description of the governance structure of the cohort, and of the opportunity to become actively involved with it. The latter should include governance bodies in which scientists and participants consider issues of common concern for the management of the cohort. For instance, issues relative to the collection, use and distribution of the data, or regarding the most appropriate informed consent mechanisms could be dealt with through direct engagement of participants. Similarly, participatory governance can promote the emergence of issues linked to the cultural diversity that is likely to characterize a very large research precision medicine cohort. This could also allow participants to steer the research ambitions of the cohort towards what are felt to be pressing public health concerns. Upon enrollment, participants should thus be made aware of the fact that – if interested – they can be involved in the activities of such bodies, directly or, in the case of large-scale cohorts, through mechanisms of representation. This exceeds what is commonly taken into account in informed consent forms and, as a matter of fact, informed consent procedures are probably ill suited for this kind of communication.

It is important, however, that those governance bodies – their composition and agendas – remain as open as possible to be determined by participants themselves. Only in this way will the existence of a participatory governance structure enable participants to form the sort of community bonds that ideals of partnership and engagement seek to promote and protect. Assuming instead that those bonds already exist and thus defining entirely in advance the available modes of participation would actually restrict the scope of ethical discussion to a limited set of pre-determined options.

The governance of scientific research is certainly one of the domains in which the value of participatory models is easier to grasp [26]. The uptake of a participatory turn in the governance of precision medicine thus represents a positive development. However, we should remain mindful of the risk that participation and partnership end up assuming an exclusively instrumental role of public legitimation [21, 32–34]. In this respect, allowing for participants to have some control on the scope and agenda of participatory governance structures shows that appeals to participation are more than just forms of political posturing.

As a matter of fact, assuming that people will be interested in active engagement in the development of personalized medicine may unnecessarily set the bar too high for the field. Recent episodes demonstrate that patients can actually interpret engagement in ways that can disenfranchise them more than making them compliant partners of the research enterprise. For instance, digital platforms such as PatientsLikeMe are often invoked as instantiations of the ideal of partnership and direct engagement in the development of personalized medicine [35]. Those platforms provide patients with the opportunity to take direct control of their role as research subjects, but they can also facilitate a form of “patient-led disobedience” (for instance, protocol violations), instead of an endorsement of the predefined aims of research [27]. Now, if we are serious about participation, we cannot fail to also recognize these more controversial incarnations of the idea of direct engagement. Using the principle of respect for autonomous agents as a normative reference point, one is in a better position to dissect the ethical value of any given form of engagement – including those that point towards forms of “disobedience” – as to their capacity to promote autonomy and expand the scope of ethically valuable interests [36, 37].

Offering such options and opportunities to perspective participants means recognizing their ethical importance as autonomous agents, that is, agents that value having those options and opportunities. Moreover, leaving it to participants to define – as far as possible – the scope of their engagement takes seriously the idea that public participation is a way to explore what participants consider common interests and common concerns. This can also help balance relations of power and control between research participants and scientific experts. Finally, providing opportunity of substantial direct engagement will have a reputational payoff for publicly-run precision medicine cohorts, thus helping to build public trust and public confidence in those initiatives.

The fulfillment of a sense of partnership can certainly constitute a stimulus for some to contribute to the development of the field by donating samples and accepting the collection of data about themselves. But most importantly, being able to nurture a sense of partnership and engagement, beyond the formalized routines of research participation, expands the array of meaningful choices available to perspective participants. According to the account of autonomy that we have presented, therefore, participants should be offered opportunities of direct engagement as a matter of respect for them as autonomous agents.

Thus far, we have shown that the value of direct engagement and participation can also be recognized if we hold respect for autonomous agents as a guiding principle in the development of medical paradigms relying on extensive datasets.

We would like to conclude by stressing, once again, that autonomy (properly understood) can actually serve to address the concerns that have animated public participation scholars for the last decade within the field of genetics, genomics and personalized medicine. A frequent worry with autonomy in this body of scholarship is that it manifests an individualistic understanding of moral life. We recognize the value of this observation. However, the Razian account of autonomy that we have advanced here clearly acknowledges that “[p]eople’s individuality expresses itself in ways fashioned by social practices, and through their ability and inclination to engage in socially formed relations and pursuits. Concern for individual freedom requires recognition that an important aspect of that ideal is the freedom of people to belong to distinctive groups, with their own beliefs and practices, and the ability of such groups to prosper” [38].

Fostering community bonds, membership and engagement is therefore more than merely compatible with having autonomy as a reference ethical principle. As a matter of fact, one of the reasons why autonomy is valuable is precisely that it allows one to choose to belong to one of those “distinctive groups”, and to engage directly with ensuring its prosperity. Far from representing two alternative ethical ideals, autonomy and partnership thus seem to entertain a far more intricate conceptual relation than has so far been imagined. With this paper, we attempted to clarify some important aspects of that relation.

## Conclusions

In current discussions and policy initiatives regarding the development of personalized medicine, the existing ethical frameworks – exemplified by the American Common Rule – is frequently called into question. Appeals to direct engagement and participation are frequent, but little theoretical work has been devoted to establishing their normative foundations yet. In this paper we have set out to fill this gap.

We have traced the emergence of precision medicine as a novel paradigm in biomedical research and clinical practice. We explained that such transformation towards increasingly personalized healthcare has proceeded alongside an equally remarkable transformation in the domain of ethics. With the growth of genetics and the advent of high-throughput sequencing technologies, the established ethical paradigm of the Belmont report and the Common Rule – centered on the value of individual choice in both research and clinical practice – started to appear inadequate. We have shown that a participatory turn – centered on the procedural notions of partnership and direct engagement – has taken shape to compensate for these perceived shortcomings. We have discussed the theoretical and practical relationships between the notion of partnership and that of autonomy and argued for a Razian understanding of the latter. More specifically, we have proposed a principle of respect for autonomous agents (or concern for autonomous lives) emphasizing the availability of meaningful arrays of choices for individuals enrolled in precision medicine cohorts. We have shown that respect for autonomous agents can vindicate the concerns raised by participatory framings of research ethics, thus providing a unified normative point of reference in this still emerging area of biomedical innovation. Finally, we have maintained that focusing on autonomy does not entail disregarding the value of communitarian bonds and activities that link a person to the communities she is a part of.

## Abbreviations

IRB: Institutional review board
PMI: Precision medicine initiative
REC: Research Ethics Committee

## Footnotes

1 Early articulations of the idea of partnership can also be found in the work of philosophers like Hans Jonas, Jay Katz and Paul Ramsey [39–42]. We thank one of the reviewers for directing our attention on this body of work.

2 In the remainder of the paper, we will use these expressions interchangeably.

3 For a more detailed account of the promise of precision medicine and of its historical antecedents, see [43].

4 The relevance of this account of autonomy for bioethics has been shown in the context of Direct-to-consumer (DTC) genomics and in the debate about the return of incidental findings in whole genome sequencing, see [14, 44].

5 Further conditions for Razian autonomy are the development of what he calls inner capacities for autonomous life, namely: the cognitive capacity to process information, the emotional capacities to make sense of the worth of our moral options, physical capacities to realize our autonomous life plans, and finally the possession of character traits enabling the pursuit of an autonomous life [31].

6 As to the other two conditions for Razian autonomy (absence of coercion and manipulation and the development of inner capacities to choose and pursue autonomously chosen values and life plans), we think they can be assumed to be implicit in the very ideal of direct engagement.

7 This entails that when we are offered opportunities to choose, whether and how this contributes to our autonomy remains to be established. Discussing in greater detail criteria of meaningfulness for options of autonomous choice in general falls beyond the scope of the present paper. For a comprehensive philosophical analysis of the topic see [45], especially chapter 14. However, in the specific field of research ethics we can rely on moral commonsense and stipulate that options are not relevant when they do not promote the interests and expectations that research participants have as they enroll in a study.
